# Examination of Symptoms of Depression among Cooperative Dairy Farmers

**DOI:** 10.3390/ijerph18073657

**Published:** 2021-04-01

**Authors:** Yanni Liang, Kai Wang, Brandi Janssen, Carri Casteel, Matthew Nonnenmann, Diane S. Rohlman

**Affiliations:** 1 Department of Occupational and Environmental Health, College of Public Health, University of Iowa, Iowa City, IA 52242, USA; brandi-janssen@uiowa.edu (B.J.); carri-casteel@uiowa.edu (C.C.); matthew-nonnenmann@uiowa.edu (M.N.); diane-rohlman@uiowa.edu (D.S.R.); 2 Department of Biostatistics, College of Public Health, University of Iowa, Iowa City, IA 52242, USA; kai-wang@uiowa.edu

**Keywords:** farmers, cooperatives, social support, service and engagement, symptoms of depression

## Abstract

Farmers experience a high risk of stress, depression, and suicide. Risk factors are well documented but protective factors are seldom examined. Social support has been reported to reduce psychological distress among the general population but its effect on farmers is inconclusive. Agricultural cooperatives are typically created and owned by farmers to secure markets, access supplies and services, and participate in decision-making. It is unknown whether having cooperative resources impacts symptoms of depression. A survey was used to examine whether having access to cooperative programs and social support impacted symptoms of depression among dairy farmers. Farm bankruptcies, stress, depression, and suicide were identified as ongoing concerns. Having social support and cooperative educational opportunities and mentorship programs were associated with decreased symptoms of depression. Conversely, having cooperative policy discussions was associated with increased symptoms of depression. Results suggest that social support can potentially reduce symptoms of depression among farmers and having access to cooperative resources can reduce or increase it, depending on the type of program. Our findings identified an opportunity to further examine how programs provided by farmer-led organizations such as cooperatives can impact stress, depression, and suicide among farmers.

## 1. Introduction

Agriculture is affected by adverse climate events, market prices, and political uncertainties, demanding workloads, and social isolation, all of which can be harmful to farmers’ mental health. Stress, depression, and suicide deaths among U.S. farmers are ongoing issues that have worsened since the 1980s farm crisis [1,2,3,4]. Farmers encounter greater risks for depression and suicide deaths compared to the general population [5,6,7]. Mental health risks among this population are well-documented (e.g., financial problems, relationship conflicts, and social isolation) [8], however, protective factors are less well known. Resources offered by farmer organizations, such as agricultural cooperatives (co-ops), and social support provided by community members may lessen the impact of stressors farmers are facing. 

Co-ops are typically created and owned by farmer-members to market products, access supplies and services, and encourage members to participate in governance [9,10]. They also may provide technical support, social interactions, and support networks for members. Co-ops first emerged in the dairy and grain sectors as farmers attempted to avoid the “middleman” and directly negotiate for market access [11,12]. Now, by aggregating resources, co-op farmers collectively process commodities, distribute finished goods, and market their products. In 2017, US agricultural co-ops generated $197 billion in total sales and represented 1,890,057 farmers (93%) nationally [13]. Farmers can join multiple co-ops and some members may be counted multiple times. Co-ops are especially common in the dairy sector, distributing more than 80% of the fluid milk produced nationwide. The effects of co-op programs on mental health have not been examined.

Research findings about the role of social support among farmers are inconclusive. Early research has shown that social support reduces psychological distress, more among younger farmers than older farmers in the US [14]. However, in another study that compared stress among farmers and non-farming rural residents, social support was only found to alleviate stress in non-farming rural residents [15]. Additional research is needed to examine whether social support availability can mitigate symptoms of depression among farmers. 

In this study, we examined whether having access to co-op programs and social support affect symptoms of depression among US Midwest farmers. 

## 2. Materials and Methods

### 2.1. Study Design

Informed by our previous qualitative research and the literature [1,8,16,17], we developed a cross-sectional study to examine associations between (a) dairy farmers’ access to co-op programs (i.e., services and engagement activities) and (b) social support availability on symptoms of depression. Our survey collected information on demographics, farming characteristics, social support availability, co-op attributes, programs offered, the number of times used, and program satisfaction during the last 12 months. The self-administered survey was designed to take 15–30 min to complete through REDCap (an online research data collection application) hosted at the University of Iowa (Vanderbilt University, Nashville, Tennessee). Participants completing the survey received a $15 check. The University of Iowa Institutional Review Board approved the study.

### 2.2. Participants and Recruitment

Participants were recruited from three Wisconsin dairy co-ops. Wisconsin was chosen as the study site because it is the second largest dairy producer nationwide and the fourth biggest co-op state by the number of headquarters [13]. Farmers who were 18 years or older and a co-op member during the last 12 months were invited to complete the survey. Co-ops assisted with participant recruitment by distributing study materials containing a cover letter describing the survey to eligible members, forwarding a link to the online survey, and sending up to two survey reminders. Co-op A mailed the study materials to 180 members and generated a 6.7% participation rate. We were unable to determine a participation response for co-ops B and C because they emailed the study materials and we did not know how many members received the emails.

### 2.3. Survey Development

Core survey items for co-op programs were developed in part from qualitative themes (i.e., stress among farmers, co-op resources, and the role of co-ops in farmers’ mental health) that we identified from our previous research (Table 1) [18,19,20]. In our previous research, we characterized farmers’ co-op participation and perceptions of co-ops in promoting mental health [17]. We interviewed co-op leaders, an agriculture educator, and farmers (N = 12) in Wisconsin, asking open-ended questions about stress among farmers, co-op structures, programs offered to and used by farmers, and the role co-ops played in promoting mental health. Concepts conveyed in the themes and participant statements relevant to the research questions formed most of the survey items. From the stress among farmers theme, we constructed questions assessing perceptions of mental health status among farmers (e.g., farmer depression and suicide) and farming outlook, and stressors (e.g., sick cows and loan difficulties) occurring during the last 12 months. The co-op resources theme was converted to questions examining attributes, services and engagement activities (i.e., programs) offered, the number of times programs were used, and program satisfaction during the last 12 months. From the role of co-ops in farmers’ mental health theme, we developed questions measuring participants’ perceptions of co-op services, engagement activities, and support networks in protecting farmers’ mental health. 

Survey items for social support was modified from the Social Support Questionnaire 6-item (SSQ 6-item) [21,22]. The SSQ 6-item assesses availability and satisfaction of perceived social support in six hypothetical events (e.g., someone to talk to and assist in a crisis) but has not been applied in studies of agricultural workers [15,23]. Therefore, we incorporated hypothetical events and language relevant to farmers [14,17,23,24]. For example, the original question, “Whom can you really count on to be dependable when you need help?” was modified to “If you need help with farm chores, who would you turn to?” Response options were either “no one” or selecting from a pre-defined list of individuals who normally interact with farmers (e.g., family, church member, and co-op staff) [14,23,24]. The survey was reviewed and pretested by five co-op and non-co-op farmers at a county fair in Southwest Wisconsin. The final survey incorporated feedback from pre-test participants.

### 2.4. Measures

The primary exposure variables were access to and satisfaction of co-op services and engagement activities. The secondary exposure variable was social support availability through co-ops, other community groups and family and friends. Perceived availability of social support was assessed because previous research indicates that this measure is strongly associated with psychological distress [25]. The outcome variable was the symptoms of depression score, which was measured by the 10-item Center for Epidemiologic Studies Depression Scale (CES-D) [26,27]. Participants were asked how often they experienced symptoms associated with depression (e.g., sleep problem and loneliness) during the past week from none of the time (0) to all of the time (3). A score above the cut-off 10 suggests a significant risk for depression [28,29]. This scale has previously been used in studies of agricultural workers [4,30,31].

### 2.5. Statistical Analysis

We administered the survey from late January to early-May 2020 (N = 49). Data with complete survey responses were analyzed (N = 45). During this period, the coronavirus disease 2019 (COVID-19) pandemic occurred worldwide. To examine the pandemic impact on survey response, we used Wisconsin’s Safer at Home Order date 24 March 2020 as a cut-off time to separate data into pre- and during-pandemic groups. Wilcoxon rank sum tests detected statistically non-significant (*p*-value > 0.05) differences in the self-report of symptoms of depression and the use of co-op programs between the pre- and during-pandemic groups. Therefore, data from both groups were analyzed together. Continuous variables for demographics and farming characteristics (e.g., age and years of farming) were dichotomized into “above” and “below” groups using a median split (e.g., > and ≤54 years). The binary variable stressor was coded into “yes” and “no” groups. The ordinal variables mental health status, farming outlook, and co-op attributes were reported as percentage distributions. The binary variable access to services and engagement activities was coded into available versus unavailable based on the responses to programs offered. Due to a small sample size, the number of times using a program and program satisfaction were not reported in this study. The social support variable was coded into available versus unavailable based on the responses to the aforementioned categories. The symptoms of depression score was treated as a continuous outcome variable. Two items assessing positive outlooks (e.g., happy and hopeful) were reverse coded. Individual participant’s CES-D score was calculated by summing up each item score and had a range of 0 to 30. Wilcoxon rank sum tests were used to compute whether symptoms of depression median scores were statistically different among participants identifying specific demographic and farming characteristics (Table 2), experiencing stressors (Table 3), having access to services (Table 4) and engagement activities (Table 5), and having social support (Table 6). The significance level selected for hypothesis tests was 0.05. The analysis was performed using SAS (version 9.4, SAS Institute, Inc., Cary, NC, USA). 

## 3. Results

### 3.1. Demographic and Operation Characteristics

Participants were primarily male (60%), married (93%), and lived with family members (91%) (Table 2). Most participants were owner-operators (98%), managed a dairy herd (67%), operated small enterprises (71% milking less than 70 cows and/or 80% farming fewer than 315 acres of field crops), and financially depended on farming (76%). The average US dairy herd size was 175 cows, and the average farm size was 441 acres in 2017 [32]. Although not statistically significant, participants reporting higher median scores on the depression symptom scale had one of these characteristics: under the median age 54, female, unmarried, relied on off-farm income, milked more than 70 cows, managed 315 or more acres, and farmed fewer than 30 years, compared to their respective counterparts. Participants living alone reported statistically significant higher median scores on the depression symptoms than participants not living alone (*p* = 0.0139).

### 3.2. Co-op Attributes

**Figure 1:**
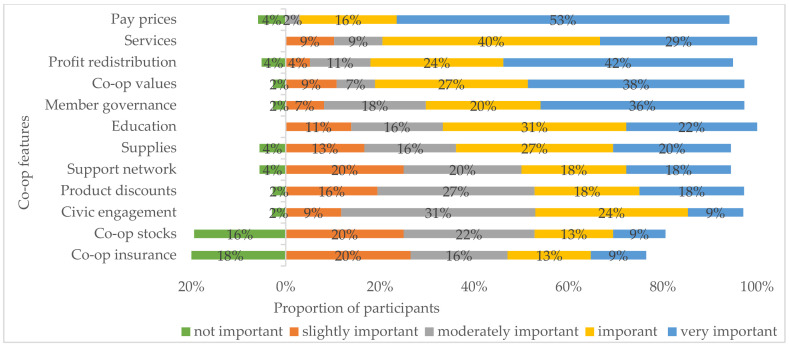
The levels of importance of co-op features from participant ranking.

Over half of the participants (58–87%) ranked all co-op attributes from slightly important to very important (on a 5-point scale from 1 = not important to 5 = very important) (Figure 1). The most valued attributes ranked as important to very important are pay prices (69%), services (69%), profit redistribution (66%), co-op values (65%), member governance (56%), and education (53%). The least valued attributes ranked as not important are member insurance (18%) and co-op stocks (16%). 

### 3.3. Mental Health Status among Farmers

**Figure 2:**
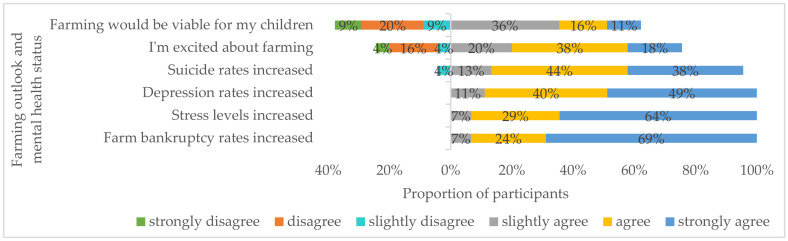
Faming outlook and mental health status among farmers in the past 12 months from participant ranking. The total ranking percentages for suicide (99%) and farming viability (101%) did not add to 100% because of rounding.

All participants had some level of agreement (from slightly agree to strongly agree) that farm bankruptcy, stress, and depression among farmers had increased over the past year (Figure 2). Most (95%) participants agreed that suicide rates among farmers had increased during the same period. Opinions on farming outlook for the present and future were diverse. While most participants were excited about farming (76%: from slightly agree to strongly agree), and viewed farming as a viable career for their children (63%: from slightly agree to strongly agree), some participants disagreed that farming was exciting (24%) or would be a viable career for their children (37%). 

### 3.4. Stressors

**Figure 3:**
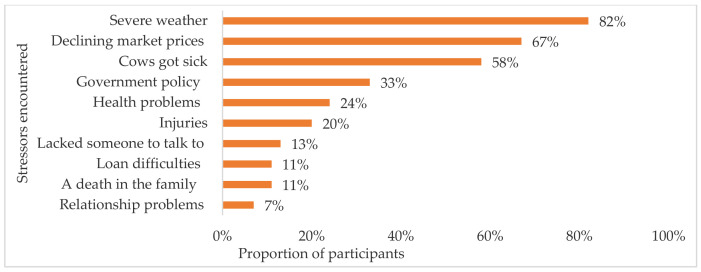
Stressors encountered by participants during the last 12 months.

Top stressors reported by over half of the participants during the last 12 months were severe weather, declining markets, and sick cows (Figure 3). Higher symptoms of depression median scores were observed among participants experiencing structural stressors like government policy negatively affecting operation, declining markets, and difficulties obtaining operating loans, occupational stressors like sick cows, individual stressors such as health problems and injuries, and relationship level stressors such as lack of someone to talk to and relationship problems (e.g., divorced or separated), compared to participants who did not face these issues (Table 3). Participants experiencing severe weather and a death in the family reported lower symptoms of depression median scores than participants not experiencing them. The score differences were statistically significant (*p* < 0.05) for most stressors, except severe weather, government policy, and health problems. 

### 3.5. Co-op Services and the Symptoms of Depression Score

**Figure 4:**
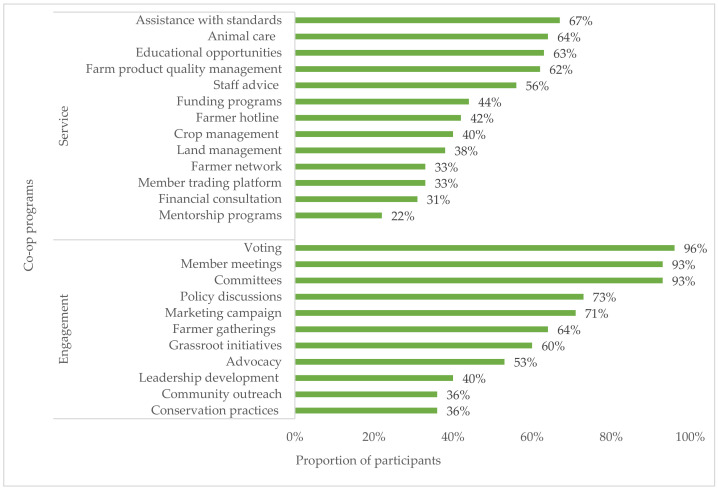
The availability of co-op service and engagement programs during the last 12 months.

The most accessible services determined by the percentage of reported availability by more than half of the participants were assistance with standards, animal care, educational opportunities, farm product quality, and staff advice (Figure 4). Participants with educational opportunities, mentorship programs, farmer network, crop management, farm product quality management, financial consultation, and land management services reported lower symptoms of depression median scores than participants not having these services (Table 4). Among these services, educational opportunities, and mentorship programs, along with staff advice were statistically significant (*p* < 0.05) with symptoms of depression scores. Conversely, participants with technical support for assistance with standards and animal care, financial services in funding programs, and general services in farmer hotlines and member trading platforms reported higher symptoms of depression median scores than participants not having these services. These services were not statistically associated with symptoms of depression (*p* > 0.05).

### 3.6. Co-op Engagement Activities and Symptoms of Depression Score

The most accessible engagement activities determined by the percentage of reported availability were voting, member meetings, committees, and policy discussions (Figure 4). Participants with member meetings, committees, marketing campaign, voting, conservation practices, community outreach, and advocacy engagement activities reported lower symptoms of depression median scores than participants not having these engagement activities (Table 5). These engagement activities were not statistically associated with symptoms of depression (*p* > 0.05). By contrast, participants with policy discussions, farmer gatherings, leadership development, and grassroot initiatives engagement activities reported higher symptoms of depression scores than participants not having these engagement activities. Only the unadjusted association between having policy discussions and symptoms of depression was statistically significant (*p* < 0.05).

### 3.7. Social Support Availability and Symptoms of Depression Score

**Figure 5:**
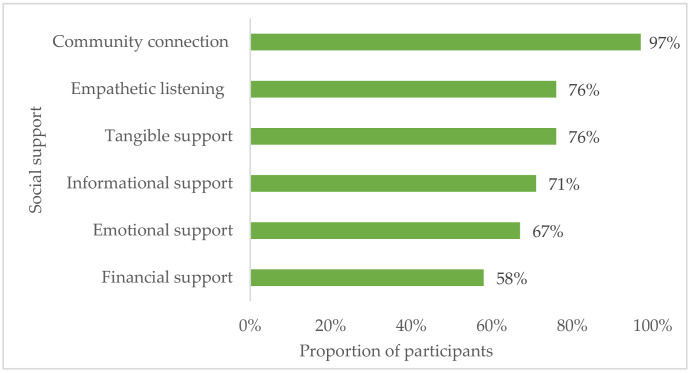
The availability of social support during the last 12 months.

Participants with emotional, informational, tangible, financial, and empathetic listening social support (Figure 5) reported lower symptoms of depression median scores than participants not having these types of social support (Table 6). Associations were statistically significant (*p* < 0.05) for emotional, informational, and tangible support only. Most (97%) participants indicated having community connections (i.e., member of other groups or organizations). 

## 4. Discussion

Co-ops empower farmers to participate in decision-making concerning markets and services [33]. Our findings demonstrated that co-ops remain economically and socially significant to farmers. Their economic influence is confirmed by the fact that over 65% of the participants identified co-op services, stable pay prices, and profit redistribution as important. Their social impact is characterized by presenting members with opportunities to align their beliefs with co-op values through participating in member governance and educational opportunities. These characteristics reflect the co-ops’ interest in promoting members’ wellbeing. 

We identified that the impact of co-op programs on symptoms of depression is specific to the types of program available. Participants having access to many of the co-op services and engagement activities reported lower symptoms of depression median scores compared to participants not having the same programs. Specifically, participants with services related to educational opportunities and mentorship programs reported fewer symptoms of depression compared to participants not having these services and the associations were statistically significant (*p* < 0.05). By contrast, participants having access to other programs (e.g., animal care, policy discussions, and leadership development) reported an increased median number of symptoms of depression compared to participants not having these programs. However, the association was only statistically significant (*p* < 0.05) for engagement activities in policy discussions. The latter observation may be due to the fact that participation in co-op policy development involves commitment of time away from farming, negotiating diverse member interests and relationships, and potential frustrations with a slow decision-making process common in democratic governance. Co-ops are governed by democratic principles where members of diverse economic interests consent on policy development or revision [33,34]. Therefore, engaging in co-op policy discussions can potentially become a source of stress. Although our unadjusted findings warrant further research, we nonetheless establish the first evidence that having specific co-op programs may have an impact on symptoms of depression. 

These quantitative results expanded our previous qualitative research by testing relationships of services and engagement activities that our interview participants perceived as important. We extended the qualitative research in three areas. First, having technical support services was not statistically associated with symptoms of depression although technical support services, including assistance with standards and animal care, were the most-sought-after. 

Second, our interview participants from the qualitative study indicated that member interaction was helpful in managing stress. However, our survey results revealed that activities that included member interactions such as farmer gatherings, marketing campaign, and community outreach were not associated with symptoms of depression (*p* > 0.05). Co-op decision-making was another important attribute highlighted in our previous research. However, activities representative of decision-making such as member meetings, committees, voting, and leadership development were not associated with symptoms of depression (*p* > 0.05). These findings could be due to the small sample size and should be further examined in larger studies. 

Finally, some interview participants from the qualitative study perceived that the role of co-ops in farmers’ mental health was conditioned on their ability to assist members obtaining operating loans. We did not observe statistically significant associations between funding programs and financial consultation and symptoms of depression. Variabilities in financial programs across co-ops may partly explain this observation, however, co-op market stability and profitability could have a greater impact on depression than influence on lending. Positive economic outlook from co-op markets may minimize the need to seek financial assistance, offset lending pressure, or instill a sense of shared uncertainties for individual farmers. Our study adds quantitative findings to the overarching objective of identifying the role of co-ops in promoting farmers’ mental health. Importantly, interpreting quantitative and qualitative results together provides us inclusive understanding of co-op impacts at individual and population levels. 

Among the six types of social support examined, five (emotional, informational, tangible, financial, and empathetic listening) were associated with having fewer symptoms of depression, but statistically significant (*p* < 0.05) associations were only found in emotional, informational, and tangible support. Farmers are less likely than the general population to discuss emotions or seek help [35], although our findings suggest that when they do, their mental health can benefit. Our previous research found that farmers were reluctant to seek help with financial problems because of concerns that neighbours may exploit a farmer’s stressful situation for their private interests. Depending on the circumstances, farmers may not seek help from neighbors or community members, but we found in this study that they seek several services from co-ops. Our previous research also suggests that co-op farmers identify co-ops as trusted sources. There is a need to further examine how farmers can use co-ops for social support.

Early mental health programs targeting farmers developed since the 1980s farm crisis have either been defunded or not evaluated for effectiveness. Recent news reports of farmer suicide redirected attention to this issue. In response, Congress has allocated funding through the FARMERS FIRST Act and the Seeding Rural Resilience Act to support states to develop stress assistance networks (e.g., counseling, support groups, and helplines) for farmers and ranchers [36,37]. While federal funding is needed, evaluating available community-based resources (e.g., co-op programs and social support) can identify context-specific intervention opportunities to address these pressing issues.

Our findings should be interpreted within study limitations. Our survey enrollment spanned from late January to early May 2020 in Wisconsin, which included 10 surveys completed after Wisconsin enacted the Safer at Home Order (24 March 2020) to reduce the COVID-19 spread. Financial and health concerns linked to the pandemic may have influenced these survey responses. For example, in April, there was news of reported milk dumping among Wisconsin farmers who lost markets. However, we concluded negligible impact after identifying no significant differences between symptoms of depression in the samples excluding and including the 10 surveys completed after the Safer at Home Order. Marketing co-ops usually renew sale agreements with members at the beginning of the year, which provides stable markets throughout the year. It was likely that our participants would have had updated their sales agreement by April. Furthermore, our participating co-ops did not rely heavily on retail outlets (e.g., schools and restaurants) to distribute products, where most market disruptions occurred. Because of the cross-sectional nature of the survey, we do not know whether the co-op services and activities used by farmers preceded symptoms of depression, or whether farmers experienced symptoms of depression and then sought out programs. Findings from our study can inform a future prospective cohort study to address this limitation.

Recruiting farmers to participate in research studies is structurally [38] and methodologically challenging [39]. Previous research conducted by our co-authors (Janssen and Nonnenmann) suggested that farmers are hesitant about participating in safety and health studies due to lack of trust and fear of adverse financial impact [40]. To encourage survey participation and completion, we excluded questions about mental health history, including a diagnosis of mental health, or information about medication because farmers are reluctant to discuss a mental health condition or obtain professional care [41,42,43,44]. Although individuals with mental health conditions are less likely to participate in survey research [45], it is possible that our sample included farmers with depression. As such, symptoms of depression may be overreported in some of our sample, potentially leading to an underestimate or overestimate of the associations observed [46]. The unexpected death of a loved one elevates the risk of depression among the general population [47], however, we observed lower depression median scores among four participants who reported a death in the family. Previous research has shown that the death of a spouse or a child was related to increased levels of stress among farmers [48], however it is not clear the impact from the death of other family members (e.g., parent, sibling). We did not know the relationships between the participants and the deceased family member, which could affect their psychological responses related to death. In addition, social support reduces depression risk [49,50,51]. Three of the four participants that lost a family member indicated having social support available, which may have contributed to their self-report of low depression scores. Our study did not include a comparison group, which may limit the generalizability of results to non-co-op farmers or farmers from different co-ops [52]. The small sample size may also have limited statistical power to detect significant associations. Farmers generally decline to participate in studies that are not related to production efficiency [40]. One co-op manager also explained to us that their members were reluctant to complete surveys. Our study presented initial evidence of co-ops’ potential influence in mental health. Further research using larger samples including co-op and non-co-op farmers is needed.

## 5. Conclusions

Having access to social support and co-op educational opportunities and mentorship programs were associated with lower median depression scores, while having opportunities to be involved in co-op policy discussions was associated with increased depression scores. Our findings suggest that, depending on the activity, co-ops can be a potential resource to support farmers’ mental health. In the absence of culturally appropriate mental health programs for farmers, further research to examine the influence of farmer-led organizations such as co-ops is important in reducing mental health risks among farmers.

## Figures and Tables

**Table 1:** Core survey items organized by themes example (see Appendix A for full table).

Themes	Core Survey Items
**Co-op Resources:** Attributes Services Engagement activities	Thinking about your experience with all the co-ops you are a member of, rank how important the following co-op attributes are to you (on a 5-point scale from 1 = not important to 5 = very important): Stable pay prices for farm product Access to affordable, quality services Access to affordable, quality farm supplies Profit redistribution Opportunities to invest in co-op stocks Member product discounts Member education Member insurance Member support network Member governance Civic engagement Sharing co-ops’ values Other (please list):

**Table 2:** Demographics and farming characteristics.

Demographics and Farming Characteristics	Median [(Interquartile Range (IQR)]	Percent (n)	Symptoms of Depression Median Score (IQR)	*p*-Value
**Age**	54 (39–61)			0.1694
≤54 years		51% (23)	9 (4–11)	
>54 years		49% (22)	5 (3–12)	
**Sex**				0.2031
Male		60% (27)	7 (4–11)	
Female		38% (17)	8 (4–11)	
Other		2% (1)	22 (22–22)	
**Marital Status**				0.1613
Married		93% (42)	7 (4–11)	
Unmarried		7% (3)	10.5 (9–12)	
**Living Situation**				**0.0139**
Alone		9% (4)	14.5 (10.5–23.5)	
Not alone		91% (41)	7 (4–10)	
**Owner-Operator**				*
Yes		98% (44)	7.5 (4.0–11.5)	
No		2% (1)	10 (10–10)	
Primary Source of Household Income **				0.1526
Farming		76% (34)	6.5 (4–11)	
Off-farm employment		22% (10)	8.5 (7–14)	
**Farm Enterprise**				0.4046
Dairy		67% (30)	7.5 (4–11)	
Non-dairy		33% (15)	8 (4–12)	
**Dairy Herd Size (head)**	70 (45–100)			0.1292
≤70		71% (32)	7 (3.5–11.0)	
>70		29% (13)	8 (5–16)	
**Field Crop Size (acres)**	315 (120–500)			0.0929
≤315		80% (36)	7 (4.0–10.5)	
>315		20% (9)	9 (8–16)	
**Years of Farming**	30 (10–38)			0.4908
≤30		58% (26)	8.5 (4–11)	
>30		42% (19)	6 (4–17)	
**Co-op Membership**				0.7908
Co-op A		31% (14)	7 (4–10)	
Co-op B		36% (16)	7.5 (4.0–10.5)	
Co-op C		13% (6)	8 (3–11)	
**Dual memberships (A and C, B and C)**		18% (8)	7.5 (3.5–12)	
Other		2% (1)	17 (17–17)	

* One participant self-reported as a non-owner operator, so p-value was not computed for owner-operator. ** One participant did not report the primary source of household income. Bold text indicates *p*-value < 0.05.

**Table 3:** Stressors and symptoms of depression scores.

Stressors Occurring during the Last 12 Months	Symptoms of Depression Median Score (Interquartile Range)	*p*-Value
Severe weather negatively affected crops or livestock		0.2758
Yes	7 (4–10)	
No	9.5 (4–16.5)	
Declining market prices negatively affected farm income		**0.037**
Yes	9 (4–16)	
No	5 (3–9)	
Sick cows		**0.0043**
Yes	9.5 (5–14)	
No	4 (3–8)	
Government policy negatively affected operation		0.1671
Yes	9 (5–11)	
No	7 (3–12)	
Health problems (e.g., chronic conditions, backpain)		0.0545
Yes	10 (6–18)	
No	7 (4–11)	
Injuries		**0.0028**
Yes	10 (10–17)	
No	5.5 (3.5–10.5)	
Lacked someone to talk to		**0.0022**
Yes	16.5 (10–21)	
No	7 (4–10)	
A death in the family		**0.0059**
Yes	2.5 (1.5–4)	
No	8 (4–12)	
Difficulties getting operating loans		**0.0062**
Yes	20 (13–26)	
No	7 (4–10)	
Relationship problems (e.g., divorced or separated)		**0.0046**
Yes	18 (17–30)	
No	7 (4–10)	

Bold text indicates *p*-value < 0.05.

**Table 4:** Associations between co-op services and symptoms of depression score.

Co-op Services	Symptoms of Depression Median Score (Interquartile Range)	*p*-Value
Educational opportunities		**0.0426**
Available	7.5 (4–11)	
Unavailable	9 (5–12)	
Mentorship programs		**0.041**
Available	7.5 (3–10)	
Unavailable	8 (4–12)	
Staff advice		**0.0423**
Available	8 (4–11)	
Unavailable	8 (4.0–11.5)	
Farmer network		0.0692
Available	5 (3–10)	
Unavailable	9 (4–12)	
Assistance with standards *		0.1101
Available	8.5 (4–14)	
Unavailable	5 (3–10)	
Crop management *		0.1145
Available	6.5 (4–11)	
Unavailable	9 (4–12)	
Farm product quality management *		0.1305
Available	7.5 (4.0–14.5)	
Unavailable	8 (5–10)	
Financial consultation **		0.1321
Available	7.5 (4–12)	
Unavailable	8 (4–11)	
Animal care *		0.2203
Available	8 (3–14)	
Unavailable	7 (5–10)	
Land management *		0.2647
Available	7 (4–14)	
Unavailable	8.5 (4.0–10.5)	
Funding programs **		0.3786
Available	10 (4–17)	
Unavailable	5.5 (3–10)	
Farmer hotline		0.3084
Available	10 (4–17)	
Unavailable	5.5 (3–10)	
Member trading platform		0.4904
Available	8 (3–11)	
Unavailable	7 (4–12)	

* Technical support services. ** Financial support services. Bold text indicates *p*-value < 0.05.

**Table 5:** Associations between co-op engagement activities and symptoms of depression score.

Co-op Engagement Activities	Symptoms of Depression Median Score (Interquartile Range)	*p*-Value
Policy discussions		**0.0325**
Available	9 (4–12)	
Unavailable	5 (3.5–9.0)	
Member meetings		0.0515
Available	7.5 (4–11)	
Unavailable	10 (5–12)	
Farmer gatherings		0.0949
Available	8 (4–11)	
Unavailable	7 (3.5–11.5)	
Marketing campaign		0.1271
Available	7 (4.0–10.5)	
Unavailable	9 (5–12)	
Committees		0.1551
Available	7.5 (4–11)	
Unavailable	10 (5–12)	
Voting		0.1207
Available	8 (4–11)	
Unavailable	8.5 (5–12)	
Conservation practices		0.2433
Available	7.5 (4.0–10.5)	
Unavailable	8 (4–12)	
Leadership development		0.2556
Available	8.5 (4–11)	
Unavailable	7 (4–12)	
Community outreach		0.2082
Available	7.5 (4–12)	
Unavailable	8 (4–11)	
Grassroot initiatives		0.3559
Available	8 (4–11)	
Unavailable	7.5 (4–12)	
Advocacy		0.4103
Available	5.5 (3.0–10.5)	
Unavailable	8 (5–12)	

Bold text indicates *p*-value < 0.05.

**Table 6:** Associations between social support availability and symptoms of depression score.

Types of Social Support	Symptoms of Depression Median Score (Interquartile Range)	*p*-Value
Emotional (when struggling)		**0.0126**
Available	6 (4–9)	
Unavailable	11 (6–17)	
Informational (questions about farm operation)		**0.0157**
Available	6 (3.5–10.0)	
Unavailable	10 (8–17)	
Tangible (help with farm chores)		**0.0237**
Available	7 (4–10)	
Unavailable	11 (6–18)	
Financial (concerns about farm finance)		0.0545
Available	7 (3–10)	
Unavailable	9 (4–17)	
Empathetic listening (someone to listen to)		0.1173
Available	7 (4–11)	
Unavailable	10 (4–16)	
Community connection (member of other groups or organizations)		0.439
Available	8 (4.0–11.5)	
Unavailable	7 (7–7)	

Bold text indicates *p*-value < 0.05.

## Data Availability

The data supporting reported results are stored at the University of Iowa and not publicly available due to privacy and confidentiality.

## Appendix A

**Table A1:** Core survey items organized by themes.

Themes	Core Survey Items
**Stress among Famers:** Farm bankruptcy Stress Depression Suicide	Rank the following statements (on a 6-point scale from 1 = strongly disagree to 6 = strongly agree): Farm bankruptcies have increased in the past year. Stress level among farmers has increased in the past year. Depression rate among farmers has increased in the last year. Suicide rate among farmers has increased in the past year. I am excited about farming. I think farming is a viable career for my children.
**Stressors:** Occupational Financial Relationship Isolation Loneliness	During the last 12 months were you in any of the following situations? (check all that apply) Cows got sick Difficulties getting operating loans A death in the family Relationship problems (e.g., divorced or separated) Health problems (e.g., chronic conditions, backpain) Injuries Lacked someone to talk to Severe weather negatively affected your crops or livestock Declining market prices negatively affected your farm income Government policy negatively affected your operation
**Co-op Services:** Advice Technical support Education/training/ mentorship Financial consultation Funding opportunities	Please indicate whether these services and resources offered by your co-ops during the last 12 months. (Services and resources include technical support, consultations and education offered by co-ops to assist members in farm practice and professional development.) Participants indicating “yes” offered were asked: Please enter the number of times you used the services and resources offered by your co-ops during the last 12 months AND rank your satisfaction. Participants entering a none zero number were asked: How satisfied are you with the _____ from your co-ops? (on a 6-point scale from 1 = strongly dissatisfied to 6 = strongly satisfied) Advice from co-op staff and or board of directors Animal care resources Crop production resources Land management resources Farm product quality management resources Standards and compliance assistance Farm financial consultations including succession planning Education programs including training and workshops Mentorship programs including apprenticeships and internships Farmer-to-farmer support network Funding opportunities including loan, grant and scholarship Member trading platform
**Co-op Engagement:** Meetings Decision-making Civic engagement Leadership Advocacy Social interactions Representation	Please indicate if these engagement activities were offered by any of the co-ops you were a member of during the last 12 months: (Engagement activities include activities and events offered or sponsored by co-ops that encourage members to participate in co-op affairs.) Participants indicating “yes” offered were asked: Please enter the number of times you participated in these engagement activities of your co-ops during the last 12 months AND rank your satisfaction. Participants entering a none zero number were asked: How satisfied are you with the _____ from your co-ops? (on a 6-point scale from 1 = strongly dissatisfied to 6 = strongly satisfied) Co-op meetings/conventions including annual, quarterly, and monthly Co-op policy and major issue discussions Co-op elections Co-op board and/or committees Grassroots movements to advocate for farmers, rural residents and consumers Advocacy groups to influence policy Farmer-led conservation initiatives Leadership development program for members Farmers’ gatherings, farm tours and leadership retreats (as an attendee or leader) Co-op sponsored community building events Co-op marketing programs (e.g., share farm stories, social media campaigns) Other (please list):
**Role of Co-ops in Farmers’ Mental Health:** Services Engagement programs Support networks	The following statements may be protective factors for farmers’ mental health. Indicate the extent to which you agree and disagree to the following conditions (on a 6-point scale from 1 = strongly disagree to 6 = strongly agree): Stable markets from co-ops Technical support from co-ops Consulting advice from co-op Positive interactions with co-op farmers Positive interactions with co-op staff/board of directors Participation in co-op decision-making process Participation in co-op marketing programs Representing co-op in public events Other (please list):
